# HBsAg Positive Patient Characteristics in Hospital and Blood Donation Camps

**DOI:** 10.1155/2013/675191

**Published:** 2013-09-04

**Authors:** Deepti Sachan, Joy Varghese, Jensingh Joseph, Vijaya Srinivasan, Venkataraman Jayanthi, Mohamed Rela

**Affiliations:** ^1^Department of Transfusion Medicine, Global Hospitals & Health City, Chennai, India; ^2^Department of Hepatology & Liver Transplantation, Global Hospitals & Health City, Chennai 600 100, India; ^3^Department of Gastroenterology & Hepatology, Kamakshi Memorial Hospital, Chennai 600 100, India

## Abstract

*Background*. Prevention of the residual risk of transfusion transmitted hepatitis B virus infection (HBV) is mostly dependant on serological screening of blood donors for HBsAg and antibody to hepatitis B core antigen (anti-HBc Ab). This study aimed to study the prevalence of HBsAg and anti-HBc Ab and to compare the profile of blood donors attending a blood donation camp and people attending a hospital based camp. *Methods*. In the blood donor camp, all the blood units were screened for HBV, (HBsAg and anti-HBc), and in the hospital based camp, screening was done for HBsAg alone. Baseline demographic characteristics were noted. *Results*. The number of blood bank donors was 363 (47.5%) and hospital camp attendees was 402 (52.5%). Prevalence of HBsAg positivity was similar in both the groups at 1.7% and 1.9%, respectively. Anti-HBc Ab positivity (Total) was 6% among the blood donors; Overall prevalence of HBV infection in this group was 3.2%. *Conclusion*. Policy for checking the collected blood unit by 3 tests for anti-HBc, anti-HBsAb, and HBsAg should be reconsidered to possibly achieve the zero risk goal of transfusion transmitted HBV infection. Blood obtained from a vaccinated donor may give an added protection to the recipient.

## 1. Introduction 

Hepatitis B virus (HBV) is one of the most common cause for chronic liver disease (CLD) in the developing countries. The virus is known to be highly infective and is associated with long-term morbidity and mortality due to complications like cirrhosis, portal hypertension, and hepatocellular carcinoma (HCC). Prevalence of hepatitis B surface antigen (HBsAg) in India varies from 1 to 13 percent, with an average of 4.7 percent [1–6]. 

Community-based prevalent studies in a selective population in Tamil Nadu state have shown prevalence of HBV infection in adults to be 27.4% and 32.7% in the younger age group (15 to 20 years) [7]. In another single community-based study from North India, consisting of 730 subjects (rural = 543; urban = 187), HBsAg was positive in 15 (2.1%) and anti-HBc in 143 (19.5%); 10 were positive for both. The overall HBV exposure rate in the population was 20.3% (148/730). The HBsAg carrier rate was similar in the urban and rural populations (1.5% and 2.3%, and not significant), and anti-HBc positivity was lower in the urban population (8.5% versus 23.3%; *P* < 0.01) [8]. 

Transmission of HBV infection through donated blood ranges from 1.2% to 1.7%. In the developing world, it is one of the major concerns in the field of transfusion medicine [9–11]. Most blood banks seldom screen for anti-HBc in the blood donors. Anti-HBc positivity indicates past HBV infection. Replacement of this blood in an immunocompromised individual can result in reactivation of the virus. 

## 2. Methods 

The World Hepatitis Day was held on 24 August 2013. As part of the program, a screening camp for HBsAg was held in the hospital campus of Dr Kamakshi Memorial Hospital, Chennai, and a blood donation camp was held at Global Health City, Chennai. 

In the blood donor camp, all the blood units were screened for HBV (HBsAg and anti-HBc), hepatitis C virus (HCV), human immunodeficiency virus (HIV) 1 and 2, HIV p24, venereal disease research laboratory (VDRL), and malaria. The data of HBsAg and anti-Hbc alone was analyzed for this study. In the hospital based camp, screening was done for HBsAg alone. 

Apart from ascertaining the prevalence of HBV infection in these two groups, we obtained further additional information on demographic profile such as age, gender, occupation, type of house, amenities in the house, previous history of jaundice or known CLD, surgery, tattoo, family history of liver disease including HCC, previous blood donations, and details of HBV vaccination. 

The data was collected in a standardized pretested proforma, by trained health workers (nurses and auxiliary health personnel). 

### 2.1. Statistical Tests

Comparison of the two groups was done using parametric tests such as chi-square, test of proportion, Student's *t*-test, analysis of variance, and parametric tests such as Wilcoxan Sign Rank Sum test.

## 3. Results 

The number of blood bank donors (BBD) was 363 (47.5%) and hospital camp attendees was 402 (52.5%). Most of the BBD participants were adults in the age range 18–58 years (median 25.5 years), and the majority were men (90.9%). In the hospital based camp (HBC) there were children (2.2%) and adults. Their age ranged from 7 to 76 years (median 23.5 years); two thirds were men (2 : 1). The age difference in the two population groups was significant with the donors being predominantly young adults (sign rank sum test *P* < 0.01). Among the BBD, one-third-were information Technology (IT) professionals holding white collar jobs (31.7%), and another one-third (37.2%), were skilled workers; college students formed 16.3%. HBC attendees were primarily hospital staff (60.5%), two-thirds of whom were auxiliary staff; doctors and nurses constituted 25.9%. The occupation of the two groups (BBD versus HBC) was significantly different (Chi square *P* < 0.001). Fifty percent of the BBD (51%) had donated blood in the past as against 7% amongst HBC attendees (*P* < 10^−5^). There was no history of drug abuse in either group. 

Previous history of surgery was significantly low amongst the BBD compared to HBC attendees (1.6% versus 5.8%) (*P* < 0.01). 

Prevalence of HBsAg positivity was similar in both the groups at 1.7% and 1.9%, respectively. Core antigen prevalence as measured by total anti-HBc positivity was 6% among the BBD. Overall prevalence of HBV infection in this group was 3.2%.

Fewer individuals were vaccinated against HBV infection in the BBD group (10.5%) compared to 27.3% among HBC attendees (*P* < 0.001). 

There was only one blood donor who had received HBV prophylaxis in the past and had an accidental needle prick prior to blood donation.

In the HBC group, vaccination rates among the hospital employees and the nonhospital attendees were similar at 28.0% and 26.3% (NS), respectively. Also, 50% of those who had donated blood in this group (HBC) in the past were protected with hepatitis B vaccine compared to those who had not donated blood (only 25.5% protected; *P* < 0.005). 

Factors such as family history of liver disease, past history of jaundice, and the number of blood transfusion received were similar in both groups.

## 4. Discussion

The present study has highlighted differences in individual characteristics between two camps: BBD and HBC, in the presence of similar HBsAg prevalence in the two groups. Anti-HBc detection in the BBD group further increased the HBV prevalence to 3.2%. Varying prevalence of antiHBc, a marker for exposure to HBV infection, has been reported from different parts of India, ranging between 8% and 18% of total donor population. In a study [12] from Behrampur, Ganjam in Orissa, 220 of 729 study population were antiHBc positive (30.1%) indicating a very high rate of exposure to HBV infection among the blood donors from this region. A significant number of them were anti-HBs positive. 

Addition of HBV DNA and nucleic acid testing (NAT) is likely to further increase the prevalence of chronic HBV infection in the community. Screening all our blood donors with all these tests is practically challenging issue. Failure to utilize this donor pool would result in further shortage of blood pool. The above tests may be appropriate in especial settings, for example, blood transfusion in an immunosuppressant or a posttransplant individual. 

The blood donors in our study were young adults, mostly males, primarily students or IT professionals or working class, with almost 50% having donated blood in the past. 

Only 10% of individuals in the blood donation camp were vaccinated against HBV. One would have expected a much larger proportion of individuals in hospital camp attendees to be vaccinated. This was not so. Only one-third of these were vaccinated. The poor vaccination status amongst young adults should be an eye opener for health authorities to undertake the task of mass vaccination campaign across the country, if one was to curtail HBV infection. There is a need for nationwide “Awareness Program” and stringent vaccination programs for both the high risk hospital professionals and also those belonging to the general population. 

## 5. Conclusion

 Policy for checking the collected blood unit by 3 tests for anti-HBc, anti-HBsAb, and HBsAg should be reconsidered to possibly achieve the zero risk goal of transfusion transmitted HBV infection. Blood obtained from a vaccinated donor may give an added protection to the recipient. 

## Abbreviations

BBD: Blood bank donors
HBsAg: Hepatitis B surface antigen
HBC: Hospital based camp
HBV: Hepatitis B virus
anti-HBc Ab: Antibody to HBcore antigen
anti-HBsAb: Anti HBs antibody
HCC: Hepatocellular carcinoma
HCV: Hepatitis C virus
HIV: Human immunodeficiency virus
HTLV: Human T-cell lymphotropic virus
VDRL: Venereal disease research laboratory.
